# The role of calcium homeostasis dysregulation in allergic rhinitis

**DOI:** 10.3389/fimmu.2026.1898643

**Published:** 2026-07-28

**Authors:** Li Xiao, Kai Chen, Yicheng Wen, Yueqi Fang, Li Tian

**Affiliations:** 1 School of Clinical Medicine, Chengdu University of Traditional Chinese Medicine, Chengdu, China; 2 Hospital of Chengdu University of Traditional Chinese Medicine, Chengdu, China

**Keywords:** allergic rhinitis, calcium homeostasis, calcium oscillations, CaSR, epigenetic regulation, NLRP3 inflammasome

## Abstract

The global prevalence of allergic rhinitis (AR) continues to rise, imposing a substantial burden on patient quality of life. Its pathogenesis is highly complex, encompassing immune dysregulation, compromised nasal mucosal epithelial barrier function, aberrant neuro-immune crosstalk, and disrupted epigenetic regulation. Current evidence suggests that dysregulation of calcium homeostasis may serve as a downstream effector and integrative regulatory node across multiple pathological pathways, thereby amplifying and sustaining AR-associated inflammation. This review systematically synthesizes the molecular evidence linking calcium homeostasis to the pathogenesis of AR. We summarize the regulatory roles of Ca^2+^ signaling in maintaining the nasal mucosal barrier, driving immune cell activation, and mediating neurogenic inflammation, alongside recent advances in understanding Ca^2+^ signaling–related epigenetic modifications in AR. Furthermore, integrating cross-disease evidence on transgenerational transmission in allergic diseases, we propose a novel scientific hypothesis: calcium homeostasis may modulate offspring susceptibility to AR via epigenetic mechanisms. This hypothesis offers new avenues for elucidating the transgenerational transmission of AR.

## Introduction

Allergic rhinitis (AR) is a chronic, non-infectious inflammatory disease of the nasal mucosa, affecting approximately 10% to 40% of the global population with rising prevalence (1, 2). In atopic individuals, exposure to allergens triggers AR, which is driven by immunoglobulin E (IgE)-mediated type 2 immunity, epithelial alarmins (including thymic stromal lymphopoietin [TSLP], interleukin-33 [IL-33], and interleukin-25 [IL-25]), group 2 innate lymphoid cells (ILC2s), and barrier dysfunction (3, 4). Beyond these classical immunological mechanisms, AR pathogenesis also involves genetic susceptibility, neuro-immune crosstalk, and gut microbiota dysbiosis; however, an integrated perspective that unifies these multifactorial contributors remains elusive (5–10).

In recent years, dysregulation of intracellular calcium (Ca^2+^) homeostasis has been increasingly recognized as a pivotal downstream effector and integrative regulatory node across multiple pathological processes in AR. It is postulated to exert potentially important regulatory effects on disease progression, symptom exacerbation, and chronic persistence. As a key second messenger, dysregulated Ca^2+^ homeostasis is involved in the regulation of multiple pathological links, including nasal mucosal barrier function, type 2 immune balance, effector cell activation, and neuro-immune crosstalk, thereby contributing to the amplification and maintenance of inflammatory responses (11–18). This review systematically synthesizes the pathogenesis of AR through the lens of calcium homeostasis and analyzes the specific roles of Ca^2+^ signaling across distinct pathological pathways. Furthermore, we preliminarily explore the potential mechanisms by which calcium homeostasis modulates transgenerational susceptibility to AR via epigenetic modifications. This work offers novel insights into the integrated multi-pathway mechanisms underlying AR and provides a theoretical reference for the subsequent exploration of therapeutic targets.

## Regulation of calcium homeostasis

In the resting state, the extracellular calcium concentration (approximately 1–2 mmol/L) is significantly higher than the cytosolic concentration (approximately 100–200 nmol/L), with over 90% of cytosolic calcium stored in intracellular calcium stores (the endoplasmic reticulum and mitochondria) (19). The maintenance of cellular calcium homeostasis is a highly complex and sophisticated physiological process that relies on the coordinated action of diverse calcium ion channels, transporters, and buffering proteins located on the membranes of organelles such as the plasma membrane, endoplasmic reticulum/sarcoplasmic reticulum, mitochondria, and lysosomes (20). Ca^2+^ oscillations are periodic, local or global fluctuations in cytosolic Ca^2+^ concentration that arise upon this homeostatic basis; rather than representing a disruption of homeostasis, they are a functional manifestation of homeostasis under dynamic regulation, achieving signal specificity through frequency encoding (21). Upon activation of cell membrane receptors, phospholipase C (PLC) hydrolyzes phosphatidylinositol 4,5-bisphosphate (PIP_2_) to generate inositol 1,4,5-trisphosphate (IP_3_) and diacylglycerol (DAG). The soluble small molecule IP_3_ diffuses into the cytosol, where it binds to and opens the inositol 1,4,5-trisphosphate receptor (IP_3_R) on the endoplasmic reticulum membrane, mediating the release of Ca^2+^ from the endoplasmic reticulum into the cytosol (21, 22). The opening of IP_3_R depends not only on IP_3_ binding but also on biphasic regulation by Ca^2+^: low-to-moderate concentrations (0.2–1.0 µM) of calcium enhance IP_3_R opening and promote calcium release via the positive feedback mechanism of calcium-induced calcium release (CICR), whereas high concentrations (>10 µM) of calcium inhibit IP_3_R via negative feedback, terminating calcium spikes and regulating the calcium oscillation cycle (23). When endoplasmic reticulum calcium stores are depleted due to sustained release, store-operated calcium entry (SOCE) is activated to replenish Ca^2+^ from the extracellular space. SOCE is primarily mediated by stromal interaction molecules (STIMs; e.g., STIM1 and STIM2) located on the endoplasmic reticulum (ER) membrane, which sense the decline in ER calcium concentration and interact with Orai calcium release-activated calcium channels (Orai channels; e.g., Orai1) on the plasma membrane, leading to Orai channel opening and facilitating extracellular Ca^2+^ influx (24–26). Following an increase in cytosolic Ca^2+^ concentration, calcium clearance mechanisms are required to restore calcium levels in preparation for the next calcium oscillation. The sarcoplasmic/endoplasmic reticulum calcium ATPase (SERCA) on the endoplasmic reticulum membrane is responsible for actively pumping cytosolic Ca^2+^ back into the ER lumen, thereby replenishing ER calcium stores (21, 27, 28). The plasma membrane calcium ATPase (PMCA) and sodium-calcium exchanger (NCX) on the plasma membrane also participate in extruding Ca^2+^ from the cell, jointly maintaining cytosolic calcium homeostasis (29–31). Mitochondria act as “rapid buffers” in the regulation of cellular calcium homeostasis. The mitochondrial calcium uniporter (MCU) on the inner mitochondrial membrane can rapidly take up calcium ions into the mitochondrial matrix when cytosolic calcium concentration rises sharply, thereby reducing the peak amplitude of cytosolic calcium signals and accelerating their decay (32). Transmembrane protein 165 (TMEM165) on the lysosomal membrane mediates Ca^2+^ transport into the lysosomal lumen in a pH-dependent manner, jointly participating in the clearance of cytosolic Ca^2+^ (33). Additionally, cytosolic calcium ion buffering proteins effectively regulate the amplitude, duration, and spatial diffusion of calcium signals through rapid, reversible binding to free Ca^2+^, thereby assisting cells in maintaining calcium homeostasis and avoiding potential calcium toxicity (34).

In addition to the core components mentioned above, various calcium channels are also involved in the regulation of calcium oscillations and the maintenance of intracellular calcium homeostasis. Among these, voltage-gated calcium channels (VGCCs) (35), ligand-gated calcium channels (LGCCs) (36, 37), transient receptor potential (TRP) channels (38), and mechanically-gated calcium channels (MGCCs) (39) represent the key pathways mediating extracellular calcium influx. Furthermore, TMEM65 is an essential component of the mitochondrial Na^+^/Ca^2+^ exchanger (mito-NCX); its depletion severely impairs mito-NCX function, whereas its overexpression enhances mitochondrial Ca^2+^ efflux (40). Zhu et al. demonstrated that activation of the lysosomal transient receptor potential mucolipin 1 (TRPML1) channel induces ER stress (41). Mallmann et al. reported that small-molecule agonists, such as TPC2-A1-N, can elevate intracellular calcium levels, although their action is independent of the two-pore channel (TPC) (42). An increase in cytosolic calcium concentration can initiate calcium oscillations by activating inositol IP_3_Rs or ryanodine receptors (RyRs) on the ER via the CICR mechanism (23, 43). Jiang et al. demonstrated that the transient receptor potential vanilloid 4 (TRPV4) channel, a mechanosensitive ion channel, responds to mechanical stress, osmotic pressure, and temperature changes, mediating calcium influx and triggering calcium oscillations (44).

## Calcium homeostasis and nasal mucosal barrier function

The nasal mucosal barrier consists of epithelial cells, tight junctions (TJs), a mucus layer, the basement membrane, and the mucociliary clearance system, which collectively mediate physical filtration, selective transcellular transport, and chemical defense (8). In patients with AR, the integrity of intercellular TJs in nasal epithelial cells is compromised, characterized by the downregulation of key TJ proteins including Claudin-1, Occludin, and Zonula occludens-1 (ZO-1), which results in increased paracellular barrier permeability (14). Mechanistically, Chang et al. demonstrated that Regulator of G-protein Signaling 1 (RGS1) mediates nasal epithelial barrier dysfunction in AR patients via modulation of the nuclear factor κB (NF-κB)/aquaporin 5 (AQP5) axis (14). In addition, RGS1 has been shown to regulate the immune phenotype of alveolar macrophage subsets by mediating intracellular Ca^2+^ responses through the PLC-IP3R signaling pathway (45). Regarding Gαi signaling, activation of G protein alpha inhibitory subunit (Gαi)-coupled receptors typically inhibits adenylyl cyclase (AC), leading to reduced intracellular cyclic adenosine monophosphate (cAMP) levels; conversely, cAMP modulates calcium channel activity via the protein kinase A (PKA) pathway (46). Ma et al. reported that RGS2 maintains intracellular Ca^2+^ homeostasis in granule cells by regulating the G protein alpha q/11 subunit (Gαq/11) signaling pathway (47). Collectively, these findings suggest that RGS1 overexpression may represent an adaptive cellular response to dysregulated Ca^2+^ homeostasis. Furthermore, the expression of protease-activated receptor 2 (PAR-2) is significantly upregulated in the nasal mucosa of AR patients. Upon stimulation of nasal mucosal epithelial cells by allergens (e.g., house dust mites) or environmental pollutants (e.g., PM2.5), PAR-2 is activated, which triggers an elevation in intracellular Ca^2+^ concentration (12, 48). Wang et al. demonstrated that PAR-2 downregulates Claudin-1 and Occludin expression, thereby inducing epithelial barrier dysfunction in AR (49).

Lee et al. demonstrated that TRPV4 overexpression in the nasal mucosa of patients with AR induces downregulation of Zonula occludens-1 (ZO-1) and Occludin expression, thereby increasing epithelial paracellular permeability (50). Interleukin-13 (IL-13), a pivotal cytokine in type 2 inflammation, has been shown to elicit inflammatory responses and epithelial barrier dysfunction in human nasal mucosal epithelial cells (51). Additionally, IL-13 has been confirmed to upregulate TRPV4 expression, exacerbating barrier disruption (50). Wang et al. reported that interleukin-1β (IL-1β) promotes the expression of matrix metalloproteinases (MMPs) by activating TRPV4 channels to trigger Ca^2+^ influx (52). MMPs can directly or indirectly interfere with the expression and localization of TJs, resulting in increased epithelial paracellular permeability and compromised barrier function (53, 54). In inflammatory skin diseases, transcriptional downregulation of STIM1 impairs SOCE and disrupts intracellular calcium homeostasis, leading to compromised TJ function (55). Phan et al. demonstrated that magnolol alleviates AR symptoms by inhibiting TRPV1 or Orai1 channels (11). Allergen exposure has been shown to enhance interleukin-33 (IL-33) secretion via the TRPV1-NF-κB pathway, thereby impairing the integrity of epithelial TJs (56). Overexpression of piezo type mechanosensitive ion channel component 1 (Piezo1), a mechanosensitive ion channel, has also been shown to induce downregulation of TJ proteins in human nasal epithelial cells, leading to increased nasal mucosal barrier permeability (57). Mucociliary clearance function may be impaired in AR patients, further reducing the efficiency of allergen clearance from the nasal cavity (8). Meanwhile, Nguyen et al. demonstrated that agonists of transient receptor potential ankyrin 1 (TRPA1) and transient receptor potential melastatin 8 (TRPM8) channels preserve mucociliary clearance function by upregulating ciliary beating in human nasal mucosa (58).

Disruption of intracellular calcium homeostasis, characterized by aberrant elevation of cytosolic Ca^2+^, precipitates mitochondrial calcium overload. Excessive mitochondrial calcium stimulates the activity of key enzymes within the tricarboxylic acid cycle (TCA cycle), augmenting electron flux through the electron transport chain (ETC). This leads to electron leakage from the ETC and the subsequent excessive generation of reactive oxygen species (ROS) (59). ROS serve as a critical driver for the translocation of high mobility group box 1 (HMGB1) from the nucleus to the cytoplasm and its eventual secretion into the extracellular milieu. HMGB1 modulates Yes-associated protein 1 (YAP) activity, and also upregulates NLRP3 expression via activation of the Toll-like receptor 4 (TLR4) pathway (60, 61). YAP exacerbates nasal mucosal epithelial barrier injury through the transforming growth factor-β (TGF-β)/Sma and Mad homolog (Smad) signaling axis (8, 62). Ma et al. demonstrated that HMGB1 compromises nasal mucosal epithelial barrier function in AR patients by promoting Bcl-2 interacting coiled-coil protein 1 (Beclin1)-mediated autophagy (63). Multiple studies have demonstrated that NLRP3-mediated pyroptosis results in the degradation of tight junction proteins and impairs the integrity of the nasal epithelial barrier (64, 65). Furthermore, ROS have been shown to directly oxidize the Fe^2+^ cofactor of prolyl hydroxylase domain-containing proteins (PHDs), thereby preventing the hydroxylation and degradation of hypoxia-inducible factor 1α (HIF-1α) (66). This mechanism drives the stable accumulation of HIF-1α even under normoxic conditions, subsequently upregulating aerobic glycolysis-related proteins such as glucose transporter 1 (GLUT1) (67). Xia et al. reported that increased GLUT1 expression promotes epithelial barrier dysfunction in AR (68). Additionally, HIF-1α has been shown to modulate epithelial barrier integrity in AR via the phosphoinositide 3-kinase (PI3K) pathway (69).

## The impact of calcium homeostasis on immune cell activation and degranulation

Ca^2+^ microdomains originate from localized, abrupt elevations in Ca^2+^ concentration driven by the opening of specific calcium channels. Channel opening has been shown to transiently elevate local Ca^2+^ levels to tens or even hundreds of micromolar concentrations; however, rapid clearance mediated by cytosolic calcium buffering systems and calcium pumps confines these high-calcium zones to a radius ranging from tens of nanometers to a few micrometers. Factors including IP3R activity, endoplasmic reticulum calcium store capacity, ion channel density, and cytosolic calcium buffering capacity collectively dictate the morphology and diffusion characteristics of Ca^2+^ microdomains (34, 70–72). Lin et al. demonstrated that the formation of Ca^2+^ nanodomains in the vicinity of calcium release-activated calcium (CRAC) channels renders cytosolic Ca^2+^ oscillations unnecessary for Ca^2+^-dependent gene expression (e.g., nuclear factor of activated T cells [NFAT]) (73). Di Capite et al. demonstrated that in mast cells (MCs) from human nasal polyp tissues, CRAC-mediated Ca^2+^ influx drives the synthesis and release of leukotriene C_4_ (LTC_4_). Via paracrine action, LTC_4_ activates the cysteinyl leukotriene type 1 receptor (CysLT_1_R) on adjacent MCs, thereby triggering further Ca^2+^ influx and forming a positive feedback loop that sustains persistent inflammation (74). Furthermore, Winterberg et al. reported that dual oxidase 2 (DUOX2) synthesizes nicotinic acid adenine dinucleotide phosphate (NAADP) and establishes Ca^2+^ microdomains within the T cell immunological synapse to initiate global Ca^2+^ signaling, and that inhibition of full DUOX2 activation reduces the production of the pro-inflammatory cytokine interleukin 17 (IL-17) by effector T cells (75).

IP3Rs are predominantly localized on the ER membrane and are activated upon binding of the intracellular second messenger IP3. Their activity is subject to biphasic regulation by cytosolic Ca^2+^ concentrations. This sophisticated gating mechanism enables IP3R activation to generate highly localized Ca^2+^ signals (23). In contrast, ryanodine receptors (RyRs) serve as the principal Ca^2+^ release channels on the sarcoplasmic reticulum (SR) and are activated via CICR. A modest influx of Ca^2+^ into the cytosol is sufficient to trigger RyR opening, thereby eliciting the release of substantial amounts of stored Ca^2+^ from the SR. This process generates more extensive, higher-amplitude, and sustained Ca^2+^ signals, commonly referred to as “calcium sparks” (76). Following the termination of Ca^2+^ signaling, calcineurin (CaN) activity decays relatively slowly. Consequently, during high-frequency Ca^2+^ oscillations, the brief intervals between Ca^2+^ peaks prevent complete inactivation of CaN, leading to cumulative elevation of its average activity and sustained dephosphorylation-dependent activation of nuclear factor of activated T cells (NFAT). Conversely, during low-frequency Ca^2+^ oscillations, the average CaN activity remains insufficient to effectively activate NFAT (77, 78). Zhai et al. demonstrated that T cells deficient in RyR2 exhibit reduced store-operated Ca^2+^ entry (SOCE) activation, resulting in delayed nuclear translocation of NFAT2, which in turn promotes differentiation toward the T helper type 1 (Th1) lineage (79). Wang et al. further reported that RyR activation mediates mast cell degranulation, leading to the release of substance P (SP) and thereby contributing to the pathogenesis of non-IgE-dependent pseudoallergic dermatitis triggered by Mas-related G protein-coupled receptor X2 (MRGPRX2) (80).

Adhesion of T cells to extracellular matrix (ECM) proteins triggers the formation of Ca^2+^ microdomains, even in the absence of T cell receptor/CD3 complex (TCR/CD3) stimulation (70, 81). These adhesion-triggered Ca^2+^ microdomains serve as initial signaling events that enhance the sensitivity of primary mouse T cells to subsequent activation (70). Zhao et al. demonstrated that substrate stiffness regulates microtubule organizing center (MTOC) reorientation and NFAT1 nuclear translocation in T cells through Piezo1 and Orai1 (82). The MTOC participates in mast cell degranulation via centrosomal microtubule nucleation (83). NFAT promotes the transcription of thymic stromal lymphopoietin (TSLP), thereby augmenting Th2 cell-mediated immune responses (84). Multiple transient receptor potential (TRP) channels have been implicated in TSLP production (84–86). Kosaka et al. reported that oxidative stress promotes the development and progression of atopic dermatitis (AD) through TRPV4-induced TSLP production (86). Elevated intracellular Ca^2+^ concentrations lead to increased mitochondrial Ca^2+^ levels, subsequently inducing hyperpolarization of the mitochondrial cristae membrane (32, 87). Chen et al. demonstrated that T helper type 0 (Th0) cells exhibiting hyperpolarized mitochondrial membrane potential (ΔΨm) display a predisposition toward differentiation into the T helper type 17 (Th17) lineage (88). However, in the RBL-2H3 mast cell line model, TRPC6 activation, while capable of eliciting cytosolic Ca^2+^ transients and promoting NFAT nuclear translocation, failed to elicit detectable degranulation responses (89). Zhu et al. demonstrated that STIM1 downregulation induces an imbalance in the Th17/regulatory T (Treg) cell ratio, characterized by upregulation of Th17 cells and downregulation of Tregs (90).

p38 mitogen-activated protein kinase (p38 MAPK) plays a pivotal regulatory role in inflammation and immune responses. Blockade of Ca^2+^ influx downregulates the phosphorylation level of p38α, whereas activation of p38α inhibits the AMP-activated protein kinase (AMPK)/mechanistic (mammalian) target of rapamycin (mTOR) pathway (84, 91–94). Tomar et al. demonstrated that inhibition of the mitochondrial calcium uniporter (MCU), which leads to elevated cytosolic Ca^2+^, enhances protein phosphatase 4 (PP4)-mediated inactivation of AMPK (95). Deficiency in AMPK signaling results in downregulation of suppressor of cytokine signaling 5 (SOCS5), impairing its ability to suppress the IL-4–STAT6–GATA3 signaling axis, thereby promoting Th2 cell differentiation (96). The activation pathway of serum and glucocorticoid-inducible kinase 1 (SGK1) depends on the PI3K/phosphatidylinositol (3,4,5)-trisphosphate (PIP3)/3-phosphoinositide-dependent protein kinase 1 (PDK1)/mechanistic target of rapamycin complex 2 (mTORC2) signaling cascade. SGK1 negatively regulates the degradation of JunB proto-oncogene, AP-1 transcription factor subunit (JunB), which is mediated by the E3 ubiquitin ligase neural precursor cell expressed developmentally downregulated 4-2 (NEDD4-2), thus promoting Th2 cell development. Mice deficient in SGK1 exhibit robust Th1-mediated immune responses (97, 98). Furthermore, SGK1 phosphorylates and inactivates forkhead box O1 (FoxO1), preventing its binding to the forkhead box P3 (Foxp3) locus and consequently suppressing regulatory T (Treg) cell differentiation (99).

In contrast, activation of the α7 nicotinic acetylcholine receptor (α7nAChR) recruits FoxO1 to the Foxp3 promoter via the IP3R/Ca^2+^/calcineurin pathway, thereby promoting Treg cell generation. Ca^2+^ signaling can also activate AMPK via the calcium/calmodulin-dependent protein kinase kinase β (CaMKKβ) pathway (100, 101). This differential regulation may be primarily driven by the frequency-dependent modulation of calcineurin (CaN) and Ca^2+^/calmodulin-dependent protein kinase II (CaMKII) by Ca^2+^ oscillations. *In vitro* reconstruction and computational modeling of neuronal synaptic plasticity have established classic frequency-dependent response zones. At low frequencies (0.1–1 Hz), calcineurin (CaN) exhibits higher affinity for calmodulin (CaM), preferentially binding CaM and exerting dominant functional activity (77, 102, 103). CaN specifically dephosphorylates phosphorylated residues on CaMKII (e.g., S1198 and S2137), thereby inhibiting its activity (104). Additionally, CaN dephosphorylates CaMKII at Thr286 via the dopamine- and cAMP-regulated phosphoprotein (DARPP-32)/protein phosphatase 1 (PP1) pathway, terminating sustained CaMKII activity (102). Conversely, at high frequencies (1–10 Hz), CaMKII undergoes autophosphorylation at Thr286. This modification significantly enhances its affinity for CaM through the “CaM trapping” effect, enabling CaMKII to continuously bind and sequester CaM. The resulting depletion of free Ca^2+^/CaM complexes suppresses CaN activity (77, 102, 103, 105). However, physiological calcium oscillations in non-excitable immune cells (e.g., T lymphocytes and mast cells) have periods ranging from tens of seconds to several minutes, corresponding to frequencies in the millihertz (mHz) to sub-Hz range (106, 107). Accordingly, calcium oscillations in immune cells can be categorized into low-frequency oscillations (0.001–0.01 Hz; periods of 100–1000 s) and high-frequency oscillations (0.01–0.1 Hz; periods of 10–100 s). Notably, the upper limit of high-frequency oscillations in immune cells (~0.1 Hz) is lower than the typical frequency threshold required for efficient CaMKII activation in neurons (~1 Hz). Nevertheless, given that CaMKII activation exhibits a cumulative frequency effect, synergistic autophosphorylation induced by multiple pulses can lower the effective frequency threshold. Furthermore, partially pre-phosphorylated CaMKII shows an approximately fourfold increase in sensitivity to low-frequency stimuli. Therefore, the possibility that high-frequency oscillations in immune cells trigger limited CaMKII activation after sufficient pulse accumulation cannot be excluded (103). Based on these premises, we hypothesize that dysregulated calcium homeostasis may induce cellular stress and mitochondrial dysfunction, resulting in insufficient intracellular ATP generation. This energy deficit fails to support high-frequency Ca^2+^ oscillations, thereby exacerbating and sustaining chronic inflammatory responses (108).

## Calcium homeostasis and neurogenic inflammation

**Figure 1:**
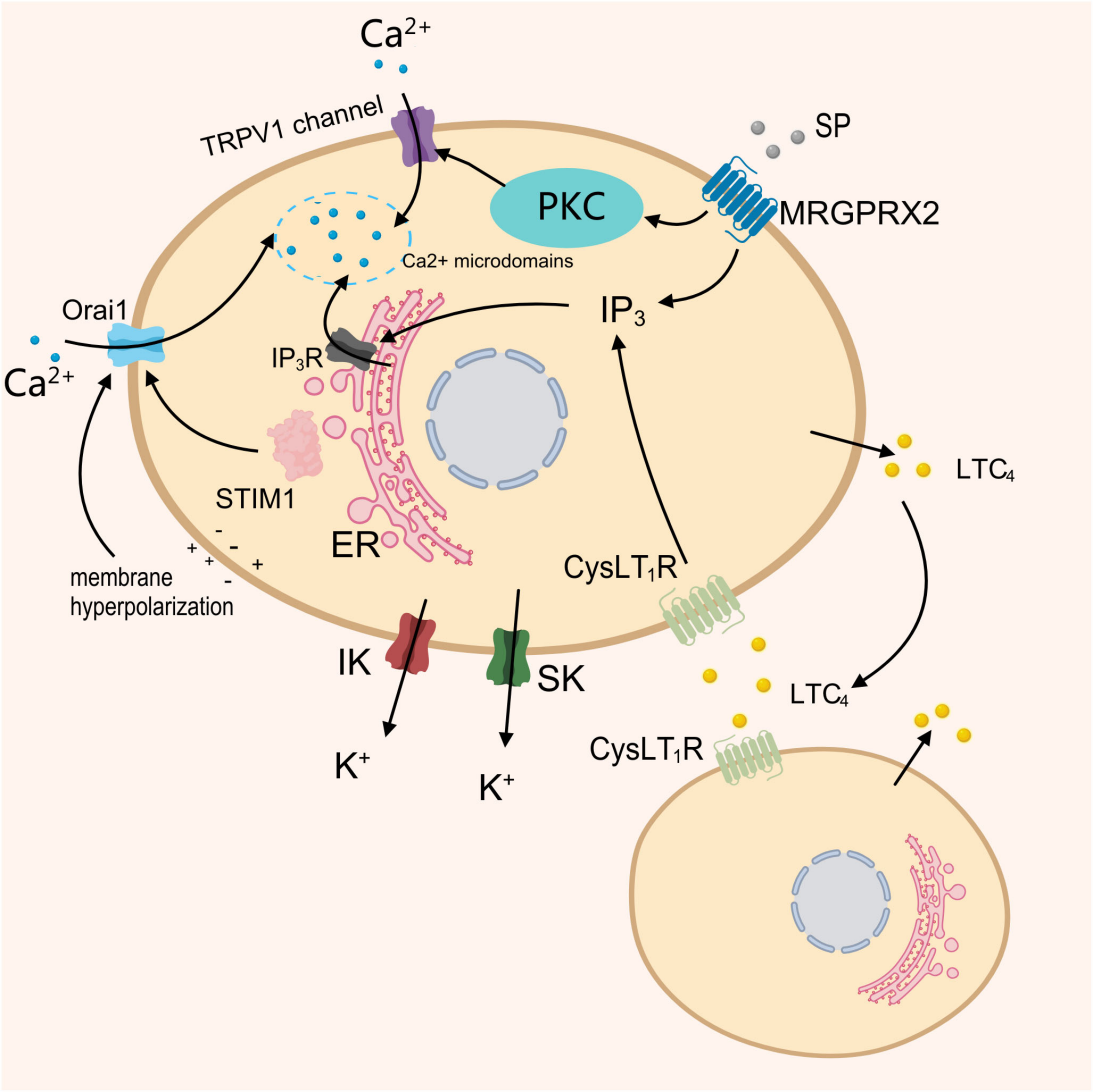
Calcium signaling pathways and positive feedback loop in mast cells. SP activates MRGPRX2 receptors on mast cells, signaling via PKC to phosphorylate TRPV1 and reduce its activation threshold. Paracrine LTC_4_ from mast cells acts on neighboring cells, signaling through GPCR-dependent PKC to similarly induce TRPV1 phosphorylation and lower its activation threshold. Elevated intracellular Ca^2+^ opens IK/SK channels, causing membrane hyperpolarization that drives sustained Ca^2+^ influx via CRAC channels and forms a positive feedback loop.

Neurogenic inflammation refers to localized inflammation initiated by the release of neuropeptides, including SP, calcitonin gene-related peptide (CGRP), and nerve growth factor (NGF), from sensory nerve terminals. In AR, these neuropeptides interact with immune cells to amplify the inflammatory cascade (6, 16, 17). An increase in intracellular Ca^2+^ concentration is a prerequisite for the exocytosis of neuropeptide-containing large dense-core vesicles (LDCVs) (109). Acetylcholine (ACh) release acts as an early event in airway epithelial cells upon airborne allergen exposure, and functions as an autocrine mediator to drive IL-33 secretion (110, 111). SP activates MRGPRX2 receptors on mast cell membranes, triggering degranulation and the release of inflammatory mediators such as histamine and tumor necrosis factor-α (TNF-α). Conversely, mast cell-derived tryptase activates PAR-2 on sensory nerve endings, to further promote SP release (6, 112). CGRP directly induces vasodilation, which contributes to nasal mucosal edema and inflammatory cell infiltration. It also upregulates SP expression and acts synergistically with SP to facilitate sensory nerve sensitization and inflammatory progression (6, 112, 113). NGF activates sensory neurons through its receptor tropomyosin receptor kinase A (TrkA), enhancing the release of neuropeptides including SP and CGRP, and thereby amplifying itch and inflammatory signaling. Proinflammatory cytokines (e.g., IL-1β) can upregulate NGF expression, forming an IL-1β/NGF positive feedback loop that exacerbates neurogenic inflammation and nasal hyperresponsiveness (6, 114). Multiple inflammatory mediators, including bradykinin, prostaglandin E_2_ (PGE_2_), and NGF, activate their respective G protein-coupled receptors (GPCRs), triggering downstream protein kinase A (PKA) and protein kinase C (PKC) signaling cascades. These pathways phosphorylate TRPV1 and TRPA1 channel proteins, thereby lowering their activation thresholds (46, 115). Upon an increase in intracellular Ca^2+^ concentration, calmodulin (CaM) pre-bound to intermediate-conductance (IK) and small-conductance (SK) Ca^2+^-activated K^+^ channels undergoes a conformational change to promote channel opening. The resulting K^+^ efflux induces membrane hyperpolarization, which in turn maintains the electrochemical driving force for CRAC channels and ensures sustained Ca^2+^ influx, a process that may ultimately drive the persistence of inflammatory responses (116–118). Consequently, Ca^2+^ channel dysfunction may sustain airway hyperresponsiveness via dysregulated calcium homeostasis, thereby exacerbating AR symptoms (**Figure 1**) (119). Recent studies have highlighted the critical role of gut microbiota in this pathological process. Li et al. demonstrated that gut microbiota dysbiosis activates the NLRP3 inflammasome, thereby exacerbating PM2.5-induced impairment of the nasal mucosal epithelial barrier (9). Following allergen-driven NLRP3 inflammasome activation via TLR4, proinflammatory cytokines such as IL-1β and IL-18 directly stimulate sensory neurons (e.g., via TRPV1 channels) to release neuropeptides, which further amplifies the inflammatory response (16, 120). Chen et al. reported that ammonia (NH_3_), a gut microbial metabolite, upregulates the expression of voltage-gated calcium channel 2.1 (CaV2.1), driving Ca^2+^ influx and enhancing ACh secretion (121). Notably, NH_3_-overproducing bacterial strains may be present in both the gut and nasal mucosa of AR patients (122). Zhou et al. further showed that ammonia inhalation alters gut microbiota composition and induces tracheal injury via TLR4 upregulation; fecal microbiota transplantation from ammonia-exposed animals to healthy recipients recapitulated this TLR4-mediated tracheal injury (123). Therefore, the intrinsic link between gut microbiota dysbiosis and neurogenic inflammation in AR patients may be associated with dysregulation of calcium homeostasis mediated by NLRP3, NH_3_, and other factors. It is important to note that this hypothesis is primarily extrapolated from studies on colonic motility. Although this mechanism is theoretically plausible—given that CaV2.1 has been established as a channel that regulates presynaptic acetylcholine release in other tissues—direct validation in the specific context of AR remains lacking (124).

## The synergistic interplay between calcium dyshomeostasis and epigenetics

Aberrant DNA methylation, specifically the methylation of cytosine at CpG sites that typically leads to gene silencing, promotes the progression of AR through two principal pathways: disrupting the Treg/Th2 balance and mediating the pathogenic effects of air pollutants (125–127). Xue et al. demonstrated that DNA methyltransferase 1 (DNMT1) exacerbates Th2 immune responses by methylating the promoter of the interleukin 12B (IL12B) gene to suppress IL-12 expression, while concurrently stabilizing T cell immunoglobulin and mucin domain containing 4 (TIM4) (128). Li et al. demonstrated that allergen exposure reduces the expression of TET methylcytosine dioxygenase 1 (TET1) in AR patients, upregulates co-stimulatory molecules (CD86, CD80, CD40) on dendritic cells (DCs), and ultimately results in excessive DC activation (129). Furthermore, Bai et al. revealed that DNMT1 regulates Foxp3 methylation, which not only restricts Treg generation during T cell differentiation, but also maintains the immunosuppressive function of mature Tregs (130). Inflammatory cytokines mediated by calcium signaling, such as IL-1β and TNF-α, can suppress TET gene expression via the NF-κB pathway (131, 132). The nasal nitric oxide (NO) concentration is significantly elevated in untreated AR patients, and NO exposure can increase DNMT activity (131, 133). The synergistic effect of TET inhibition and DNMT activation leads to aberrant DNA methylation in the promoter regions of pro-inflammatory genes such as IL6, resulting in excessive immune cell activation (134). The enzymatic activity of TET proteins is directly dependent on the availability of α-ketoglutarate (α-KG), Fe^2+^, and oxygen (O_2_). As a key intermediate of the tricarboxylic acid (TCA) cycle, the abundance of α-KG directly links cellular metabolic status to the epigenetic regulation of the genome (135). Previous studies have established multiple Ca^2+^ signaling–related epigenetic regulatory pathways in non-AR systems. For instance, Cegarra et al. demonstrated that Ca^2+^ can act as a non-competitive inhibitor to reversibly block divalent metal transporter 1 (DMT1)-mediated Fe^2+^ uptake, thereby reducing intracellular Fe^2+^ accumulation (136). Adenosine 5′-monophosphate-activated protein kinase (AMPK) activation directly phosphorylates TET2 at the serine 99 (Ser-99) site; this phosphorylation protects TET2 from dephosphorylation by protein phosphatase 2A (PP2A) and subsequent proteasomal degradation via binding to 14-3–3 proteins (137, 138). The enhanced stability of phosphorylated TET2 enables it to target specific chromatin regions and catalyze the conversion of 5-methylcytosine (5mC) to 5-hydroxymethylcytosine (5hmC), ultimately promoting DNA demethylation (139). In addition, AMPK activation can enhance mitochondrial biogenesis and improve mitochondrial quality control, thereby increasing α-KG levels (140). Wei et al. demonstrated that protein kinase B (AKT) phosphorylates DNMT1 at the S143 site. This modification blocks SET domain containing 7 (SET7)-mediated methylation at the adjacent K142 site, thereby inhibiting the ubiquitination and degradation of DNMT1 (141). It is important to clarify that the aforementioned regulatory mechanisms have been established exclusively in non-AR models; thus, their presence and functional necessity in AR pathogenesis currently lack direct experimental evidence. To date, the existing direct evidence in the AR field stems from the study by Tan et al., which demonstrated that TET2 downregulation in CD4^+^ T cells derived from AR settings leads to a genome-wide reduction in 5-hydroxymethylcytosine (5hmC) levels and aberrant hydroxymethylation profiles. This disruption consequently breaks the Th1/Th2 and Treg/Th17 immune balance, thereby exacerbating AR inflammation (142). Building upon these findings, we hypothesize that dysregulated calcium homeostasis may contribute to immune imbalance in AR by modulating multiple signaling pathways, including p38α/AMPK/mTOR and NF-κB, to mediate aberrant DNA methylation.

Histone methylation levels are dynamically orchestrated by the opposing actions of histone methyltransferases (HMTs) and histone demethylases (HDMs). HMTs rely on S-adenosylmethionine (SAM) as the primary methyl donor; consequently, intracellular SAM concentrations directly dictate their catalytic efficiency. Conversely, the SAM metabolite S-adenosylhomocysteine (SAH) acts as a potent inhibitor of HMT activity. In contrast, HDMs, particularly the JmjC domain-containing family, require α-KG as an essential cofactor for their catalytic function (143). Trimethylation of lysine 4 on histone H3 (H3K4me3) serves as a canonical hallmark of active gene transcription, facilitating the assembly of the pre-initiation complex (144). Yu et al. demonstrated that the c-Myc proto-oncogene/methionine adenosyltransferase 2A (c-Myc/MAT2A) axis regulates SAM biosynthesis, thereby mediating the upregulation of SET and MYND domain-containing protein 3 (SMYD3)-dependent H3K4me3 modifications and ultimately promoting Th0-to-Th2 differentiation (145). Furthermore, Wu et al. reported that H3K27me3 hypermethylation, mediated by the histone methyltransferase EZH2 and associated with NF-κB p65, may contribute to the development of wheezing following pulmonary inflammation (146). In the context of cancer research, basal c-MYC expression is dependent on the mTORC1 pathway; aberrant activation of the Ca^2+^/calcineurin (CaN)/NFATc1 signaling axis can additionally drive its overexpression (147–149). Collectively, these findings suggest that calcium signaling may contribute to the pathogenesis of AR by modulating histone methylation; however, the complete pathway has yet to be directly validated in the context of AR.

Histone acetylation levels are co-regulated by histone acetyltransferases (HATs) and histone deacetylases (HDACs). Histone deacetylase 1 (HDAC1) is upregulated in the nasal mucosa of patients with AR and promotes the release of Th2-type inflammatory cytokines (e.g., IL-4, IL-13) by suppressing anti-inflammatory gene transcription (150). Filaggrin (FLG) is a key protein for maintaining nasal mucosal barrier integrity, and its deficient expression leads to impaired barrier function. Histone acetylation modification bidirectionally regulates FLG expression: TNF-α/interferon-γ (IFN-γ) suppresses FLG expression via the Fos-related antigen 1 (FRA1)/c-Jun proto-oncogene (c-Jun)/histone deacetylase 1 (HDAC1) complex, while overexpression of the HAT family member E1A binding protein p300 (p300) enhances FLG promoter activity (150–152). At the cellular and molecular level, CaMKIV phosphorylates CREB at Ser133, inducing a conformational change that exposes the binding interface for the KIX domain of CBP/p300. This interaction recruits the histone acetyltransferase (HAT) domain of CBP/p300 to histones—particularly H3K27 acetylation (H3K27ac)—ultimately relaxing chromatin conformation to activate gene transcription (153, 154). Furthermore, CaMKII/IV phosphorylates specific serine residues on HDAC4 (e.g., S246, S467, S632) and HDAC5 (e.g., S259, S498), promoting their binding to 14-3–3 proteins. This interaction masks the nuclear localization signal (NLS) while exposing the nuclear export signal (NES), culminating in nucleocytoplasmic shuttling via the chromosomal region maintenance 1 (CRM1)-dependent pathway (155, 156). The enzymatic activity of SIRT1, a class III HDAC, strictly requires NAD^+^ as a cofactor. An elevated NAD^+^/NADH ratio enhances SIRT1 activity, whereas a decreased ratio suppresses it. Mitochondrial dysfunction, such as impaired oxidative phosphorylation, leads to NADH accumulation and a reduced NAD^+^/NADH ratio, thereby inhibiting SIRT1 activity (140, 157, 158). Schader et al. demonstrated that overexpression of NADPH oxidase 4 (Nox4) upregulates HDAC4 phosphorylation at Ser632 and promotes its nuclear export via ROS-mediated oxidative modification of the protein (159). Notably, disrupted calcium homeostasis directly impairs mitochondrial function and triggers excessive ROS production (59). Although these regulatory paradigms have been well-established in various cellular systems, their precise roles in the pathogenesis of AR remain to be elucidated.

In T cells, previous studies have firmly established that calcineurin-mediated concerted dephosphorylation at multiple sites triggers an all-or-none nuclear translocation of NFATc2. Once in the nucleus, NFATc2 serves as an essential regulatory factor for the assembly of the transcriptional activation complex at the IL-4 locus. Its chromatin binding is tightly coupled with the recruitment of epigenetic regulators, including BRG1 (Brahma-related gene 1), the catalytic subunit of the SWI/SNF (SWItch/Sucrose Non-Fermentable) chromatin remodeling complex, and the coactivator p300. Together, they drive the formation of a fully functional transcriptional activation complex to initiate IL-4 expression (160). Under normal physiological conditions, NFAT1 mediates a negative feedback loop to restrict sustained IL-4 expression during the late phase of the response by promoting HDAC1 enrichment at the IL-4 promoter to exert transcriptional repression. In the absence of NFAT1, this late-phase negative regulation is abolished, resulting in a persistently permissive chromatin state at the IL-4 promoter, as evidenced by increased chromatin accessibility, enrichment of active histone modifications, and DNA hypomethylation. Within this open chromatin architecture, JUNB binding to the IL-4 promoter is significantly enhanced. Meanwhile, special AT-rich sequence-binding protein 1 (SATB1), as a global chromatin organizer, acts synergistically with JUNB to augment IL-4 transcription, compensatorily driving sustained, high-level IL-4 expression (161). Collectively, the seemingly contradictory observations that “NFAT is an essential factor for IL-4 expression” and “NFAT1 deficiency leads to IL-4 overexpression” actually reflect the functional dichotomy of the same regulatory network during the acute phase (transcriptional initiation) and chronic phase (loss of negative feedback) of inflammation. This biphasic regulatory pattern aligns well with the aforementioned core logic of calcium signaling in regulating histone modifications and chromatin states, thus providing corroborating evidence for the overarching hypothesis that calcium signaling contributes to AR-associated Th2 inflammation via epigenetic modifications.

There is a complex crosstalk between non-coding RNAs (ncRNAs) and histone modifications. Long non-coding RNAs (lncRNAs) can function as scaffolds or guides, recruiting histone-modifying enzymes to specific genomic loci to regulate gene expression (125, 162). Yang et al. demonstrated that the lncRNA HOXA-AS2 directly interacts with the Polycomb Repressive Complex 2 (PRC2) to modulate histone methylation at the peroxisome proliferator-activated receptor gamma coactivator 1-alpha (PGC-1α) promoter (163). Zhan et al. reported that methyltransferase-like 3 (METTL3)-mediated m6A modification of circular RNA CDKAL1 (circCDKAL1) facilitates its nuclear-to-cytoplasmic translocation via YTH domain-containing protein 1 (YTHDC1). In the cytoplasm, circCDKAL1 interacts with insulin-like growth factor 2 mRNA-binding protein 2 (IGF2BP2) to regulate the Jumonji and AT-rich interaction domain-containing protein 2 (JARID2)/HMGB1 axis, thereby promoting M1 macrophage polarization and impairing the nasal mucosal epithelial barrier function in AR patients (164). In the context of AR, various ncRNAs are dysregulated and closely associated with disease pathogenesis (165, 166). Recent studies indicate that miR-155 is downregulated while BTB and CNC homolog 1 (BACH1) is upregulated in the nasal mucosa of AR patients. Overexpression of miR-155 regulates the Th1/Th2 balance by suppressing BACH1, which leads to the activation of the nuclear factor E2-related factor 2 (Nrf2)/heme oxygenase 1 (HO-1) pathway (167). Additionally, circCramp1l interacts with miR-532-3p to relieve its inhibition of HMGB1. HMGB1 then binds directly to dynamin-related protein 1 (Drp1), promoting Drp1 phosphorylation at Ser616. This induces excessive mitochondrial fission and ROS accumulation, activating the purinergic receptor P2X7 (P2X7R)/TLR4/NLRP3 signaling axis and ultimately driving Th2 polarization (7). Furthermore, the lncRNA PELATON competitively binds miR-10b-5p to upregulate GATA3 expression, leading to Th2 hyperactivation in AR (168). Calcium homeostasis imbalance regulates the expression, stability, and function of ncRNAs via autophagy and the ubiquitin-proteasome system (UPS), thereby participating in various physiological and pathological processes (41, 169, 170). Multiple studies have identified that the E3 ubiquitin ligase ZSWIM8 (zinc finger SWIM domain-containing 8) specifically ubiquitinates miRNAs by recognizing distinct Argonaute (AGO) proteins and RNA conformations, resulting in targeted miRNA degradation (TDMD) (171, 172). When RNA-binding proteins (RBPs) are degraded, the stability and function of their bound ncRNAs are compromised. Moreover, autophagy can recognize and degrade ubiquitinated RBP-ncRNA complexes via selective autophagy receptors (e.g., p62/SQSTM1), thereby affecting miRNA levels and activity (173, 174). When intracellular ncRNA degradation pathways are defective, ncRNAs may be packaged into extracellular vesicles (EVs) for secretion. These vesicular ncRNAs can interact with Toll-like receptors (TLRs) through various mechanisms to modulate immune responses (175, 176). Aberrant Ca^2+^ influx in the resting state can lead to cellular stress, mitochondrial damage, and impaired ATP generation, which is critical for the ubiquitination process (108, 177). Based on these findings, we hypothesize that impaired ATP generation resulting from disrupted calcium homeostasis promotes the extracellular secretion of ncRNAs via modulating ubiquitination. The secreted ncRNAs subsequently activate TLR signaling pathways, thereby sustaining chronic inflammation. Importantly, however, this deduction is currently based on indirect evidence and lacks experimental validation. Furthermore, there is a paucity of data supporting the specific role and pathological significance of this mechanism in the context of AR.

## CaSR regulates systemic cellular calcium homeostasis

The calcium-sensing receptor (CaSR) is expressed on various immune cells, including T cells, macrophages, and neutrophils. Its activation promotes the release of pro-inflammatory cytokines such as IL-6 and TNF-α, triggers NLRP3 inflammasome activation, and mediates the chemotactic migration of monocytes to inflammatory sites, thereby participating in the innate immune response. Conversely, pro-inflammatory cytokines like IL-6 can further upregulate CaSR expression, establishing a positive feedback loop that amplifies inflammation (132, 178–180). Multiple studies have demonstrated that CaSR is highly expressed in the airway smooth muscle and immune cells of asthmatic patients, and its activation is closely associated with airway hyperresponsiveness (AHR) and inflammation (181, 182).

Approximately 98% of filtered calcium is reabsorbed within the nephron, a process in which CaSR, expressed throughout the renal tubules, plays a critical role (183, 184). When serum calcium levels rise, CaSR in the thick ascending limb of the loop of Henle (TAL) is activated. This activation inhibits the renal outer medullary potassium channel (ROMK) and the Na^+^-K^+^-2Cl^-^ cotransporter 2 (NKCC2), leading to a reduction in the lumen-positive potential. Consequently, paracellular calcium reabsorption is diminished, resulting in increased urinary calcium excretion (184, 185). Furthermore, CaSR expressed on the basolateral membrane of the distal convoluted tubule (DCT) can directly influence calcium transport by inhibiting the activity of the inwardly rectifying potassium channel 4.1/5.1 heterotetramer (Kir4.1/Kir5.1) (184, 186). In addition to these direct renal effects, CaSR indirectly regulates renal calcium reabsorption by modulating the secretion of parathyroid hormone (PTH) (187).

The activation of CaSR exhibits significant cell-type and tissue specificity. Through downstream signaling pathways such as PLC and PKC, it can promote TRPC3/TRPC6-mediated calcium influx in glomerular mesangial cells. In the renal distal convoluted tubule/connecting tubule, it bidirectionally regulates TRPV5-mediated calcium reabsorption depending on its membrane localization. In contrast, it inhibits TRPV6-mediated intestinal calcium absorption in the gut epithelium (188, 189). Chen et al. demonstrated that CaSR and TRPV4 can be coupled via the phospholipase A2/cytochrome P450 (PLA2/CYP450) and PLC/PKC pathways to promote Ca^2+^-dependent M1 macrophage polarization (190). CaSR is tuned via inter-subunit disulfide bonds to respond to Ca^2+^ with a pEC_50_ in the range of 2.2–2.6 mM, which corresponds precisely to the normal physiological range of human serum calcium concentration (191). Certain L-amino acids, particularly aromatic ones like L-tryptophan (L-Trp) and L-phenylalanine (L-Phe), can act as positive allosteric modulators of CaSR, increasing its sensitivity to Ca^2+^ and lowering its EC_50_ value (192). Matarage et al. demonstrated that caffeine can also act as a positive allosteric modulator of CaSR, and that typical caffeine consumption can easily achieve plasma concentrations effective for CaSR activation (<10 μM) (193). Multiple studies have demonstrated that vitamin D exerts fine-tuned control over serum calcium levels through a complex negative feedback loop with parathyroid hormone (PTH) and synergistic interactions with other nutrients (194, 195). Epidemiological evidence indicates a clear correlation between vitamin D insufficiency and both the incidence and severity of AR. Furthermore, vitamin D supplementation may alleviate AR-associated clinical symptoms by modulating the Th1/Th2 immune balance, suppressing eosinophilic inflammation, and enhancing mucosal barrier function (196, 197). Building on a comprehensive synthesis of the preceding evidence and logical extrapolation, we propose the following scientific hypothesis: CaSR may serve as a molecular sentinel that detects physiological fluctuations in blood calcium concentrations—even those within the normal reference range. By differentially regulating Ca^2+^ channels across diverse systemic cell types, CaSR could perturb local and systemic cellular calcium homeostasis, thereby acting as one of the upstream drivers in the pathogenesis and progression of AR. Notably, direct experimental evidence supporting this hypothesis in the context of AR is currently lacking. While existing research on airway CaSR has predominantly focused on bronchial asthma, this hypothesis receives indirect theoretical support from the “united airway disease” concept linking upper and lower airway inflammation. Additionally, the potential molecular basis underlying the regulatory effects of different dietary patterns on gut microbiota composition and chronic inflammatory states may involve the modulation of CaSR sensitivity, which could subsequently exert biological effects via calcium homeostatic pathways (198–200).

Recent large-scale genome-wide association studies (GWAS), Mendelian randomization analyses, and multi-omics integration studies have established that genetic factors significantly modulate the risk of AR. Furthermore, AR shares a common genetic basis with other allergic diseases, including asthma, atopic dermatitis, and food allergies (10, 201, 202). Epigenetic mechanisms are also closely linked to the transgenerational inheritance of AR and the increased disease risk in offspring. Environmental exposures can regulate gene expression without altering the underlying DNA sequence through epigenetic modifications such as DNA methylation, histone modifications, and non-coding RNAs, thereby transmitting parental allergic susceptibility to subsequent generations (203, 204). Although extensive epigenetic reprogramming occurs during gametogenesis and early embryonic development to erase most acquired marks, certain specific DNA methylation regions, histone variants, and small non-coding RNAs (sncRNAs) carried by sperm can escape this resetting process (205, 206). Takahashi et al. (207) demonstrated, using a mouse model engineered via targeted DNA methylation editing, that acquired DNA methylation at promoter-associated CpG islands can be stably inherited across multiple generations through both paternal and maternal germlines. This transgenerational transmission, accompanied by target gene silencing and metabolic phenotypes such as obesity and hypercholesterolemia, does not rely on DNA sequence alterations. Instead, it is achieved through a reconstructable epigenetic memory that re-establishes methylation marks post-implantation. Furthermore, paternal pre-pregnancy lifestyle and dietary habits can transmit environmentally induced epigenetic information to offspring without changing the DNA sequence. This occurs via remodeling the DNA methylation landscape and sncRNA cargo in sperm, which can interfere with early embryonic development and placentation, exerting long-term effects on the immune programming of the offspring (208, 209). During mammalian fertilization and early embryonic development, the pattern of calcium oscillations is jointly determined by sperm-specific factors, endogenous oocyte signaling pathways, organelle functional status, and external environmental conditions. Aberrant calcium oscillations not only lead to fertilization failure or early embryonic arrest but may also affect postnatal physiological homeostasis through epigenetic reprogramming and metabolic imprinting (210–212). Building on the aforementioned evidence, we hypothesize that parental factors, such as dietary habits and environmental exposures, may perturb systemic cellular calcium homeostasis by affecting the functional sensitivity of CaSR. We further speculate that this calcium-homeostasis-driven mode of epigenetic regulation may be transmitted via sperm and oocytes to the zygote, where it could modulate epigenetic reprogramming in a comparable manner, thereby potentially increasing the risk of AR in the offspring of affected individuals (142, 213). Current research estimates the heritability of AR to range from 33% to 91%. Gene-environment interactions may represent one explanatory mechanism for this “missing heritability,” as the impact of certain genetic variants on AR risk may be modified by specific environmental exposures (202). While there is clear biological plausibility for the role of vitamin D in regulating fetal immune system development, multiple large-scale randomized controlled trials (RCTs) and their long-term follow-up studies have not consistently demonstrated that prenatal vitamin D supplementation reduces the incidence of childhood AR (196, 197, 214). The aforementioned hypothesis based on calcium homeostasis may offer a molecular explanation for this paradox: inter-individual variations in diet and environment may lead to heterogeneity in CaSR sensitivity, which may attenuate the expected biological effects of uniform-dose vitamin D supplementation.

## Concluding remarks

The pathogenesis of AR is highly complex and multifactorial. Its pathological progression encompasses multiple pathological dimensions and regulatory pathways, including immune imbalance, nasal mucosal epithelial barrier dysfunction, dysregulated neuro-immune crosstalk, gut microbiota dysbiosis, and aberrant epigenetic modifications. Dysregulated calcium homeostasis may serve as a convergent integrative node linking these multifaceted pathological alterations. However, notable gaps remain in our understanding of the regulatory network of calcium signaling in AR. The functional roles of certain pathways are currently extrapolated from studies on other airway diseases, such as asthma, and there is still a paucity of direct, AR-specific evidence. Furthermore, the maintenance of calcium homeostasis relies on a systemic, multi-targeted, and multi-tiered regulatory mechanism, making it difficult to achieve effective systemic intervention merely through the isolated agonism or antagonism of a single calcium channel. Therefore, further AR-specific basic and clinical studies are warranted to comprehensively elucidate the intricate regulatory network of calcium homeostasis and on this basis explore precision intervention strategies that may offer novel therapeutic directions for AR management.
